# Supplementary material for: Structural analysis of HTL and D14 proteins reveals the basis for ligand selectivity in Striga
Source: Nat Commun. 2018 Sep 26;9:3947. doi: 10.1038/s41467-018-06452-2 (PMC6158167; doi:10.1038/s41467-018-06452-2)
Supplement: Supplementary file 1 — Supplementary Information [file 41467_2018_6452_MOESM1_ESM.pdf]

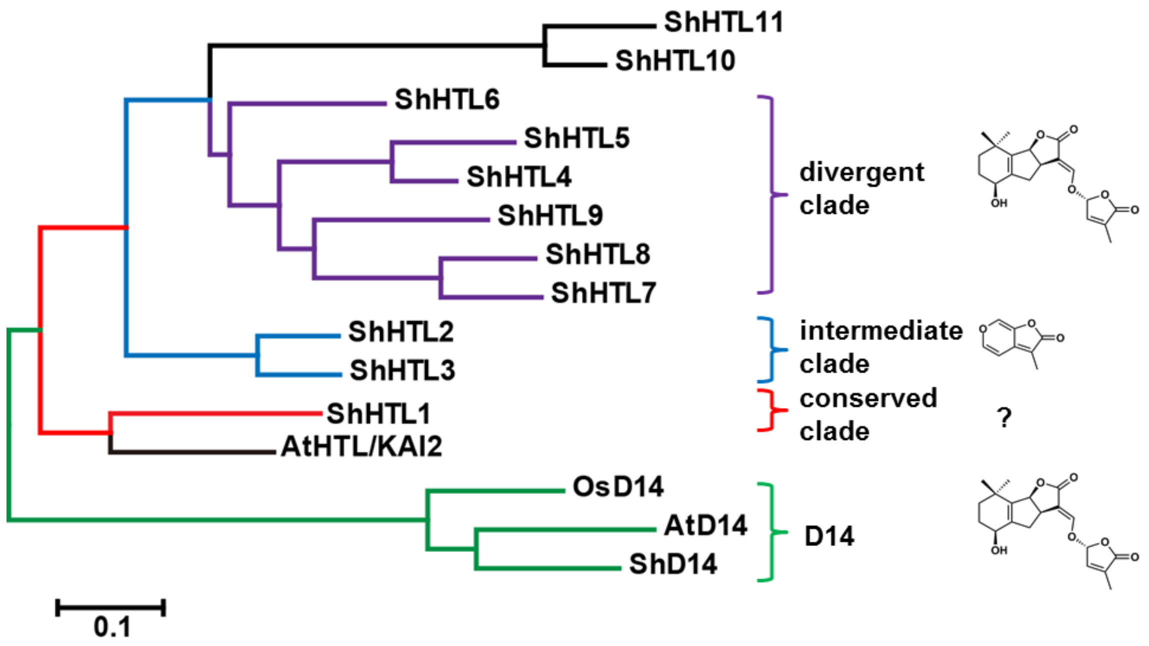

Supplementary Figure 1. Phylogenetic tree of the HTL and D14 family. An UPGMA (unweighted pair-group method with arithmetic mean) phylogenetic tree was generated by MEGA 6.0[1] from the amino acid sequences of the D14 and HTL proteins from *Oryza sativa*, *Arabidopsis thaliana* and *Striga hermonthica*. The clades of conserved, intermediate and divergent HTLs are colored red, blue and purple, respectively. ShHTL10 and ShHTL11 have been classified into a different group from ShHTL4–9 because of their inactivity in *htl*-complementary assays[2]. The D14 clade is colored green. The reported corresponding ligands[3] are depicted on the right. The branch length represents the number of substitutions per site.

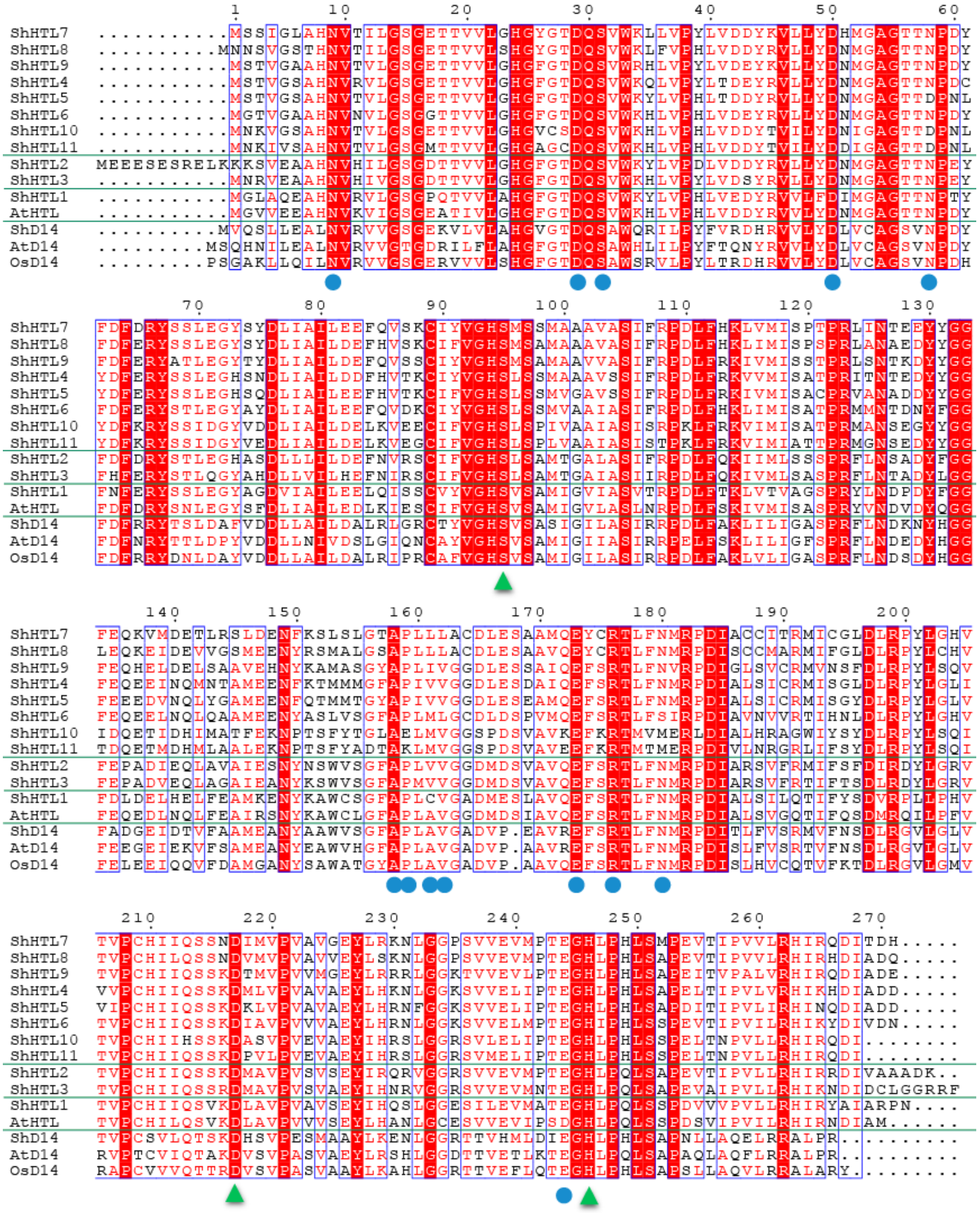

Supplementary Figure 2. Multiple sequence alignment of HTL/D14 proteins. Identical residues are shaded red, and homologous residues are shown as red letters. The residue number of ShHTL7 is shown at the top of the alignment. Catalytic triad residues are highlighted with green triangles, and residues involved in D3-interaction are highlighted with blue dots. Green lines delimit D14 and the individual clades of HTLs. Sequence alignments were performed by CLUSTALW[4] with default parameters, and the results were displayed using ESPript 3.0[5].

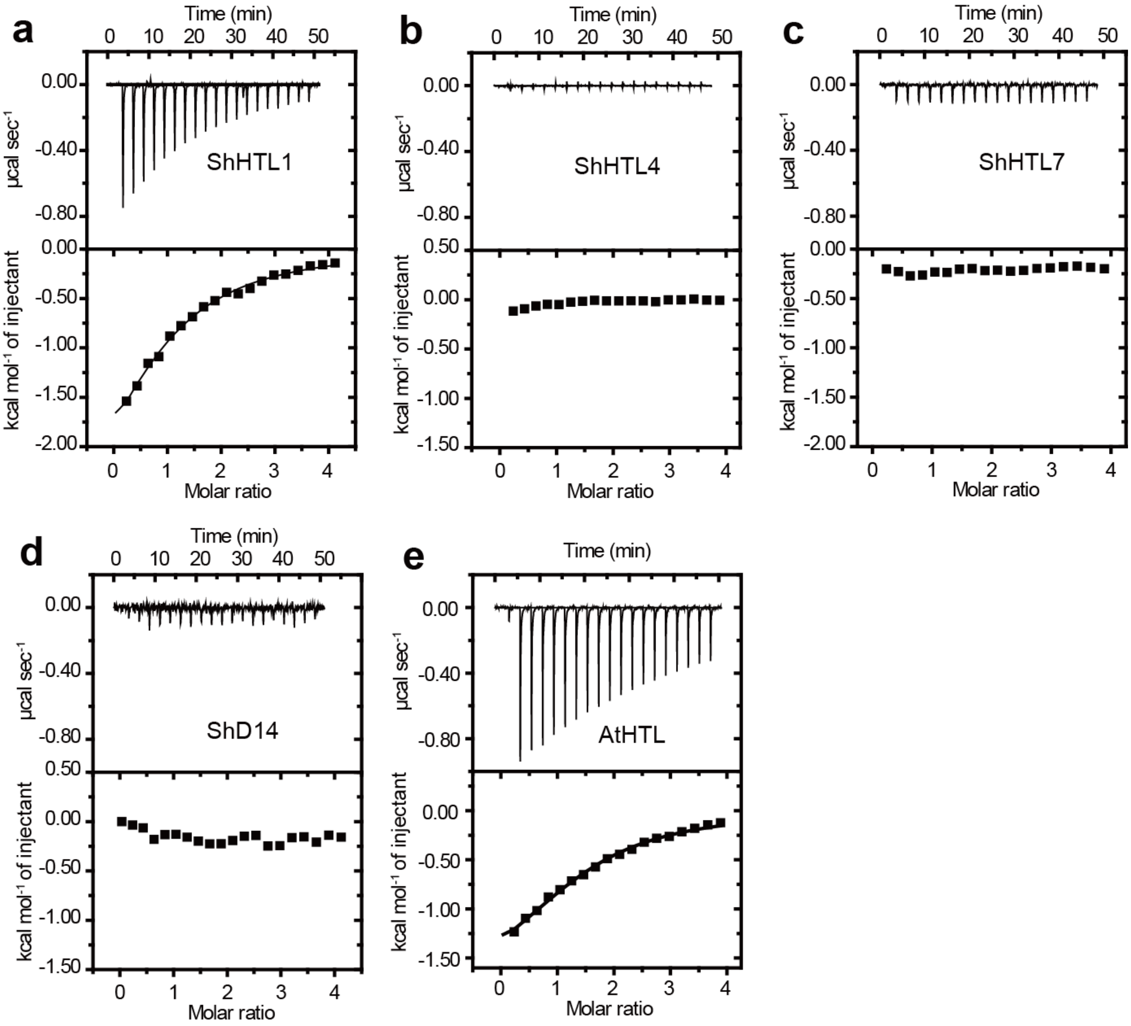

Supplementary Figure 3. Binding analyses of ShHTLs and ShD14 with KAR_1. The ITC assays were performed by adding 3 mM KAR_1 to 150 µM ShHTL1 (**a**), ShHTL4 (**b**), ShHTL7 (**c**), ShD14 (**d**) and AtHTL (**e**). The upper panel for each experiment shows the ITC titration curves, and the bottom panel shows the integrated heats of injection (black squares). Data for ShHTL1 and AtHTL were fitted using the MicroCal origin software with the "one binding site" model. The ITC assays titrating KAR_1 into ShHTL1 and AtHTL were repeated three times independently (data not shown).

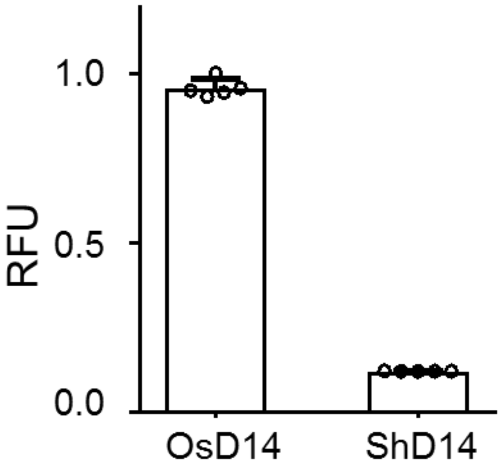

Supplementary Figure 4. YLG hydrolysis assays of OsD14 and ShD14. RFU (relative fluorescence units) was calculated by dividing the fluorescence of OsD14. The fluorescence can be detected under a 490 nm excitation wavelength and a 520 nm emission wavelength if YLG is hydrolyzed. The experiments were repeated 4 (OsD14) or 6 times independently. Columns represent the mean RFU, and the error bars represent the standard deviation (S.D.). Each replicate is indicated by a black circle.

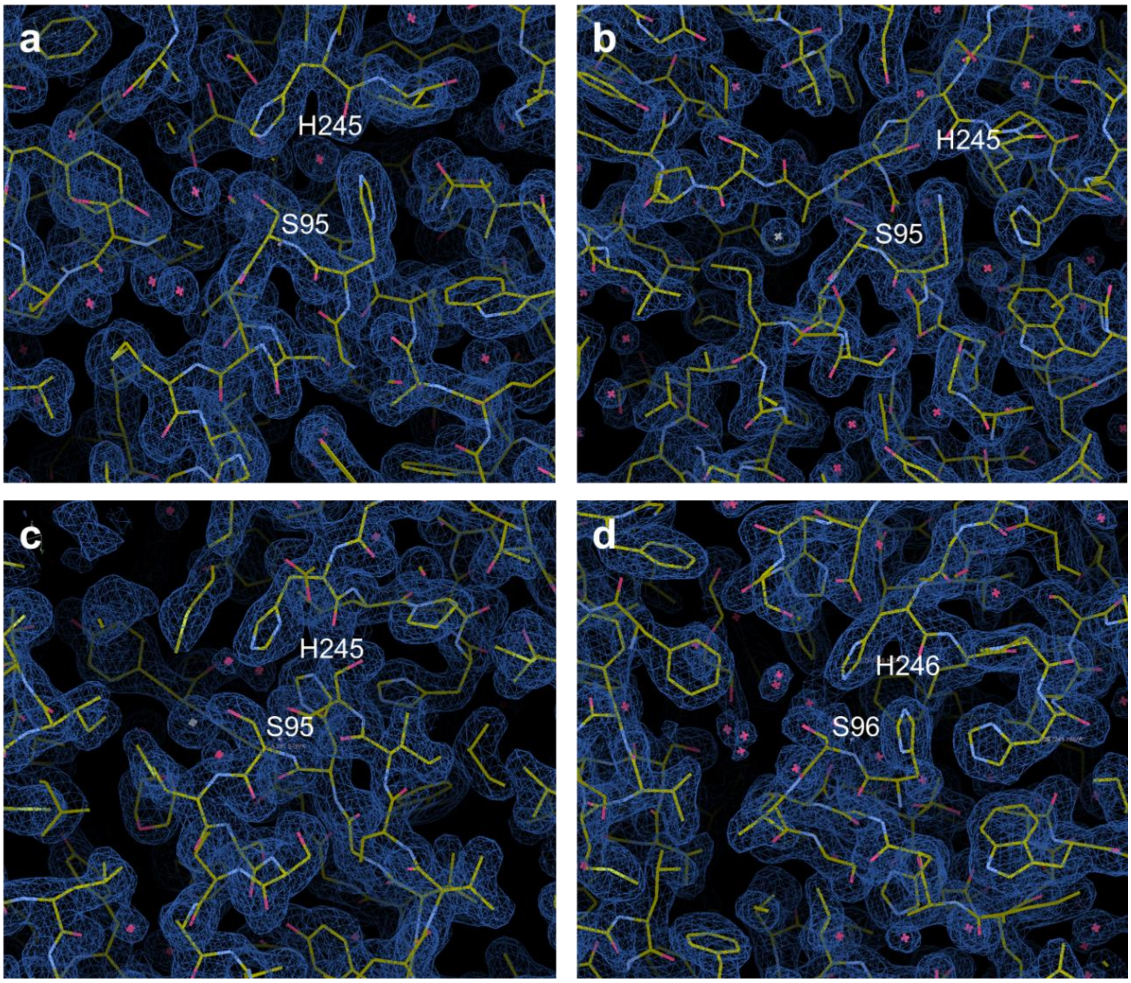

Supplementary Figure 5. Electron density maps of ShHTLs and ShD14. 2F_o-Fc electron density maps of the ligand-binding pocket of ShHTL1 (**a**), ShHTL4 (**b**), ShHTL7 (**c**) and ShD14 (**d**) are shown (blue; contoured at the 1.0σ level). Catalytic residues S95 ($S96^{ShD14}$) and H245 ($H246^{ShD14}$) are indicated.

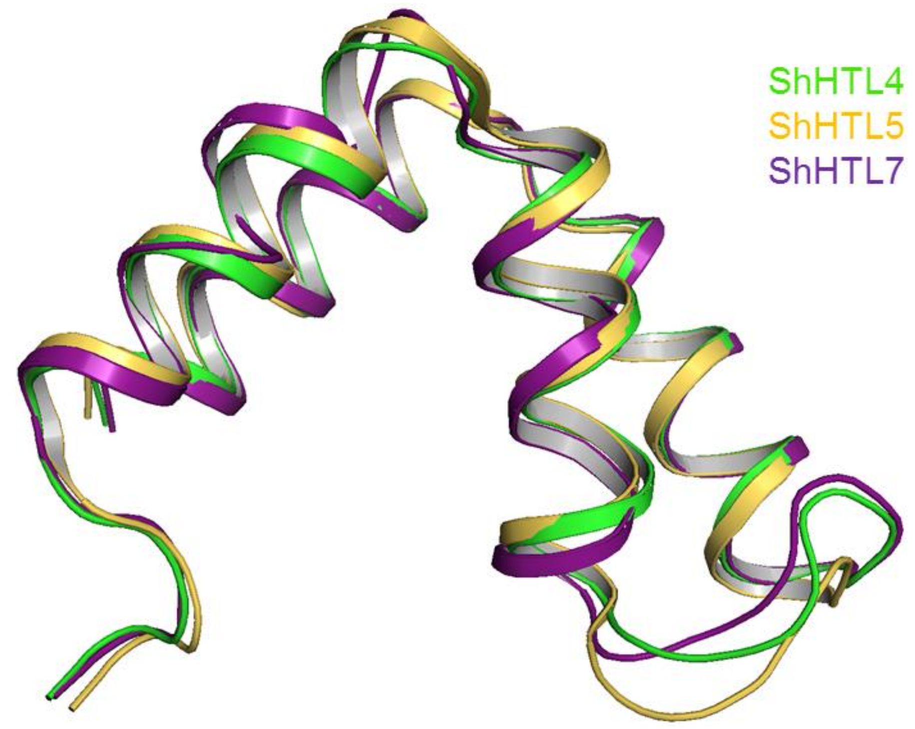

Supplementary Figure 6. Structural alignment of the cap domains. ShHTL4, ShHTL5 (PDB code: 5CBK) and ShHTL7 are shown in green, yellow and purple, respectively.

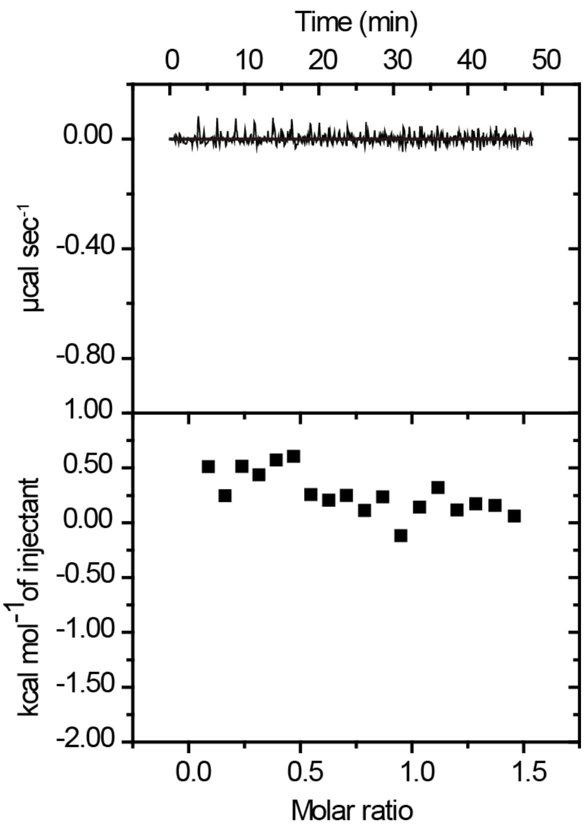

Supplementary Figure 7. ITC binding curves for the titration of ShHTL1 into *rac*-GR24. The ITC assays were performed by adding 750 µM ShHTL1 to 100 µM *rac*-GR24 at 20 °C. There was no detectable binding of *rac*-GR24 to ShHTL1. The upper panel shows the ITC titration curves, and the bottom panel shows the integrated heats of injection (black squares).

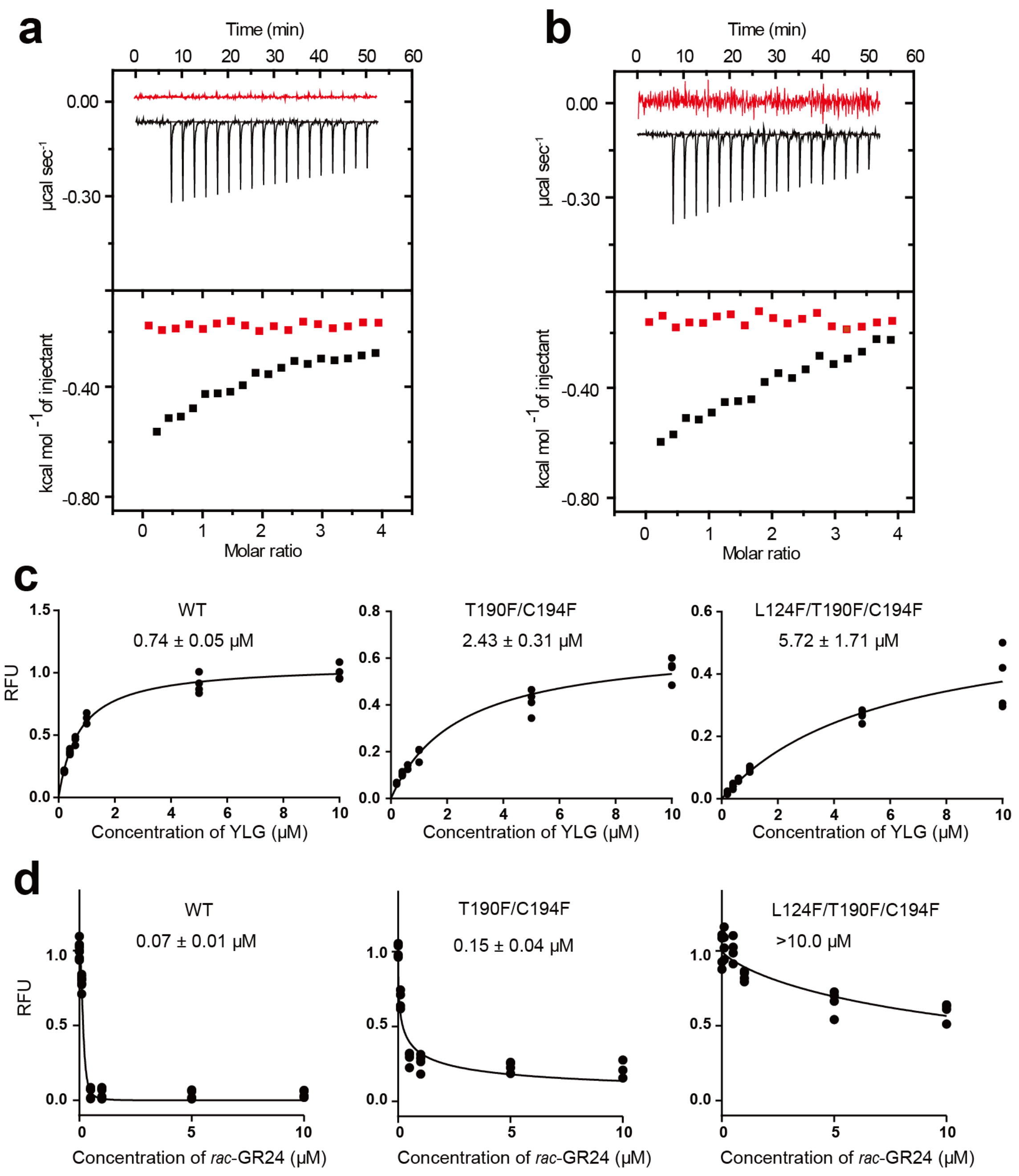

Supplementary Figure 8. Activity measurements of ShHTL7 mutants. (**a-b**), Binding analyses of the T190F/C194F double mutation (**a**) and L124F/T190F/C194F triple mutation (**b**) of ShHTL7 with KAR_1 by ITC (shown in black). The ITC assays were performed by adding 3 mM KAR_1 to 150 µM ShHTL7 mutants in the same manner as in Supplementary Figure 3. Results of titrating buffer to 150 µM ShHTL7 mutants are shown in red. The upper panel for each experiment shows the ITC titration curves, and the bottom panel shows the integrated heats of injection (black squares). (**c**) YLG hydrolytic activity of ShHTL7 and its mutants. As described

in a previous study[6], the enzymes were incubated with YLG at the indicated concentrations. The RFU (relative fluorescence units) was calculated by dividing the fluorescence of hydrolysis at 10 μM YLG by wild type (WT) ShHTL7 after subtracting the fluorescence of the buffer. The K_m values were calculated from a Michaelis-Menten plot using GraphPad Prism software and are shown in the plots. (**d**) Competition assays with synthetic SL *rac*-GR24. WT and ShHTL7 protein mutants were incubated with 1 μM YLG and *rac*-GR24 at different concentrations. The RFU was determined relative to the fluorescence values at 0 μM *rac*-GR24 and IC_{50} was calculated with GraphPad Prism software. IC_{50} values were 0.17 ± 0.01 μM for WT; 0.21 ± 0.05 μM for the T190F/C194F double mutation; and 13.92 ± 3.41 μM for the L124F/T190F/C194F triple mutation. The inhibition constant K_i was calculated according to the Cheng-Prusoff equation[7]. $K_i^{rac\text{-GR24}}$ values were 0.07 ± 0.01 μM for WT; 0.15 ± 0.04 μM for the T190F/C194F double mutation; and 11.85 ± 3.43 μM for the L124F/T190F/C194F triple mutation as shown in the plots. The experiment was repeated 4 times independently, and each replicate is represented by a black dot. The mean values are fitted, and the error bars represent the standard deviation (S.D.) of three replicates (means ± S.D., n=3).

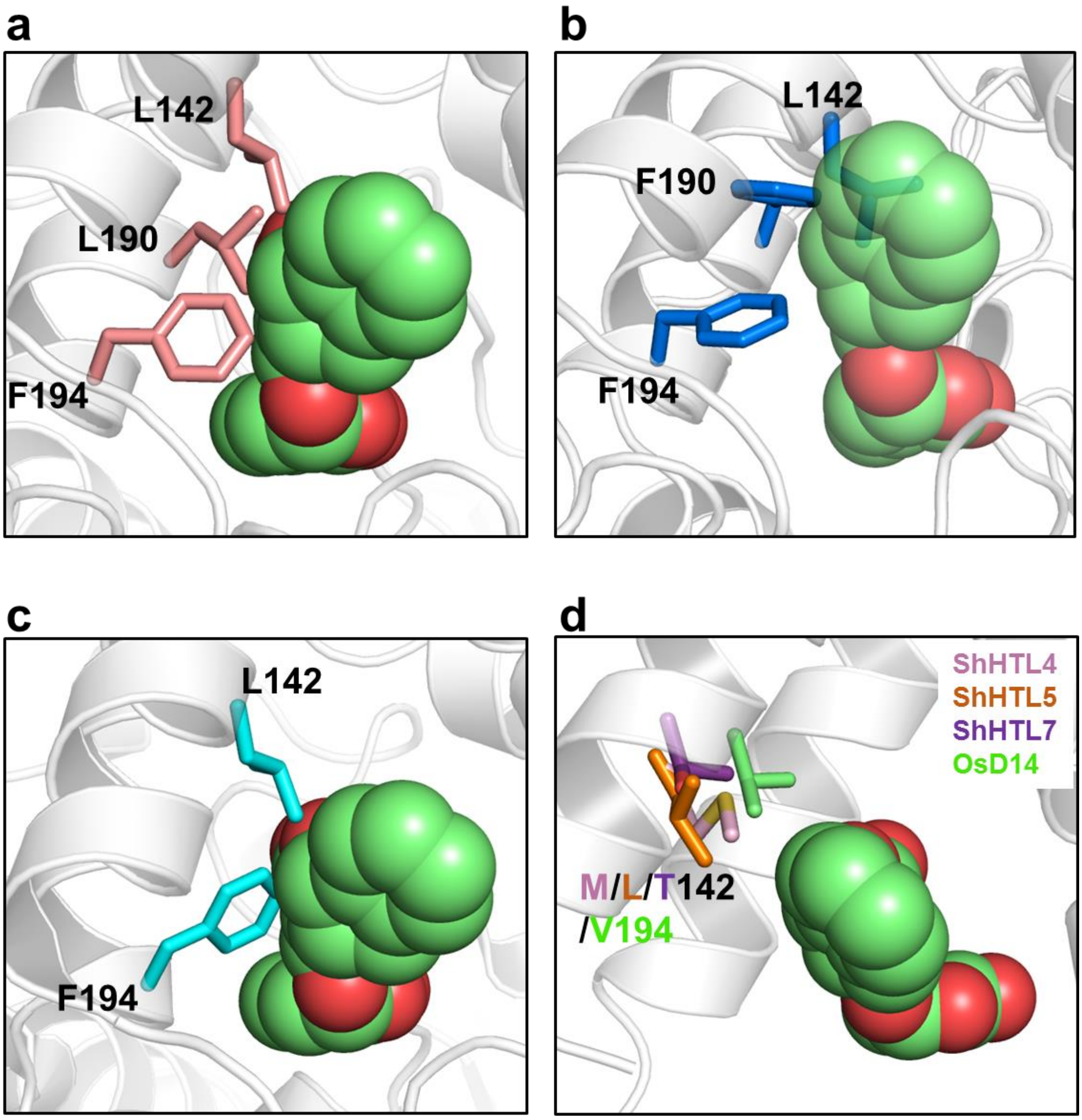

Supplementary Figure 9. Structural overlay of GR24^{5DS}-bound OsD14 with ShHTLs and ShD14. (**a**–**c**) Structural overlay of GR24^{5DS} from the OsD14-GR24^{5DS} complex with ShHTL1 (**a**), ShHTL3 (**b**, PDB code: 5DNU; blue) and AtHTL (**c**, PDB code: 4JYP; cyan) to show steric hindrance. (**d**) Structural overlay of GR24^{5DS} from the OsD14-GR24^{5DS} complex (PDB code: 5DJ5; green) with ShHTL4 (pink), ShHTL5 (PDB code: 5CBK; orange) and ShHTL7 (purple). GR24^{5DS} is shown in a sphere representation (green), and residues 142 are shown in a stick representation.

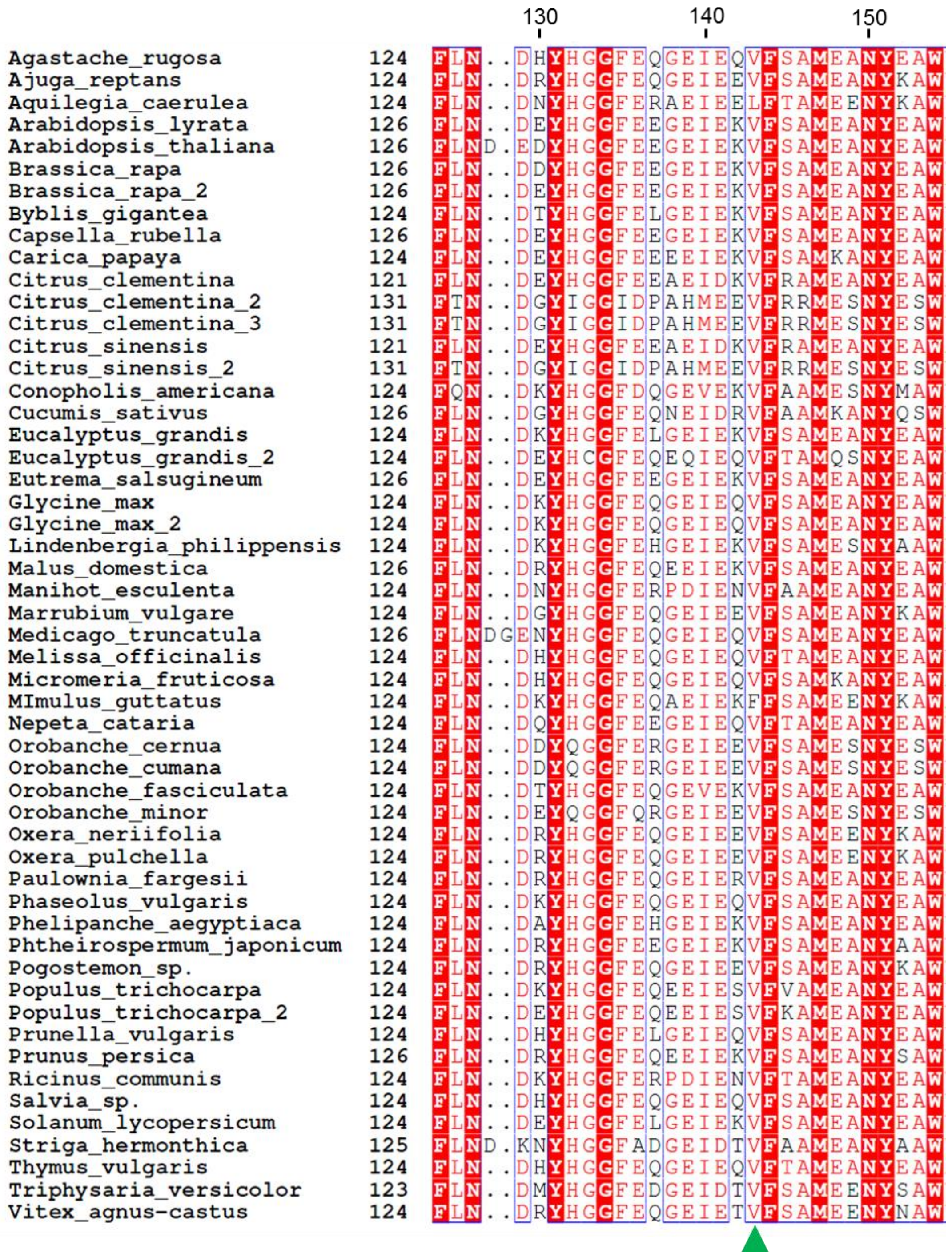

Supplementary Figure 10. Multiple sequence alignment of D14 proteins. Identical residues are shaded red, and homologous residues are shown as red letters. The residue numbers shown at the top of the alignment refer to those of ShD14. Residue 143 of D14 is highlighted with a green triangle. Sequences of D14 proteins are from both parasitic and nonparasitic plants as described previously[3]. Species names are used as sequence names. Alignments were performed by CLUSTALW[4] with the default parameters, and the results were displayed using ESPript 3.0[5].

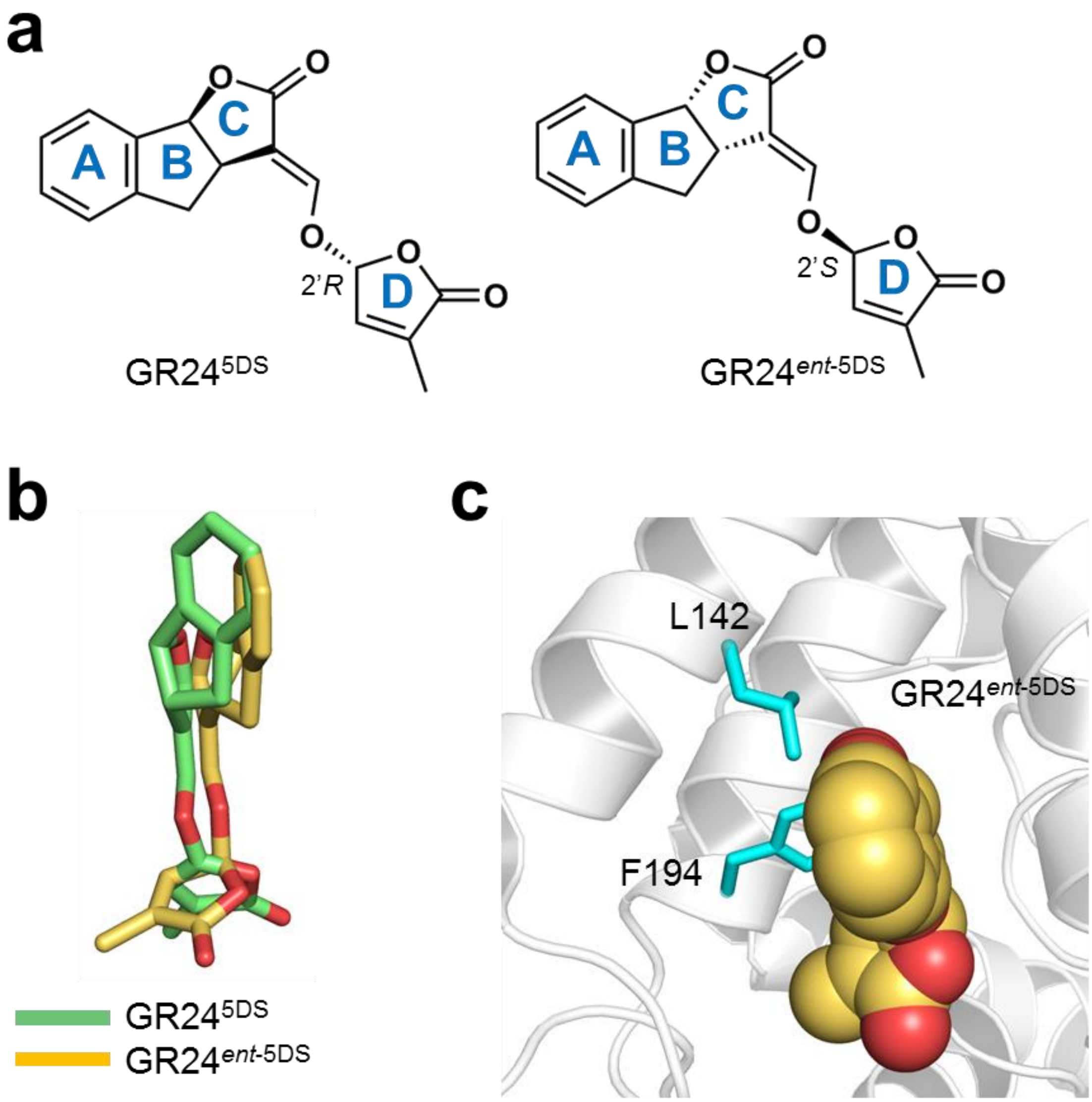

Supplementary Figure 11. Modeled structure of GR24$^{ent\text{-}5DS}$ in the pocket of AtHTL. (**a**) The structures of GR24^{5DS} and GR24$^{ent\text{-}5DS}$ are shown. (**b**) Alignment of GR24^{5DS} and GR24$^{ent\text{-}5DS}$ was generated with D rings of GR24^{5DS} and GR24$^{ent\text{-}5DS}$ on the opposite side with minor manual adjustment. GR24^{5DS} is shown in green and GR24$^{ent\text{-}5DS}$ in yellow. (**c**) The modeled GR24$^{ent\text{-}5DS}$ was docked into the pocket of AtHTL manually. GR24$^{ent\text{-}5DS}$ is presented as yellow spheres, and L142 and F194 of AtHTL are presented as cyan sticks.

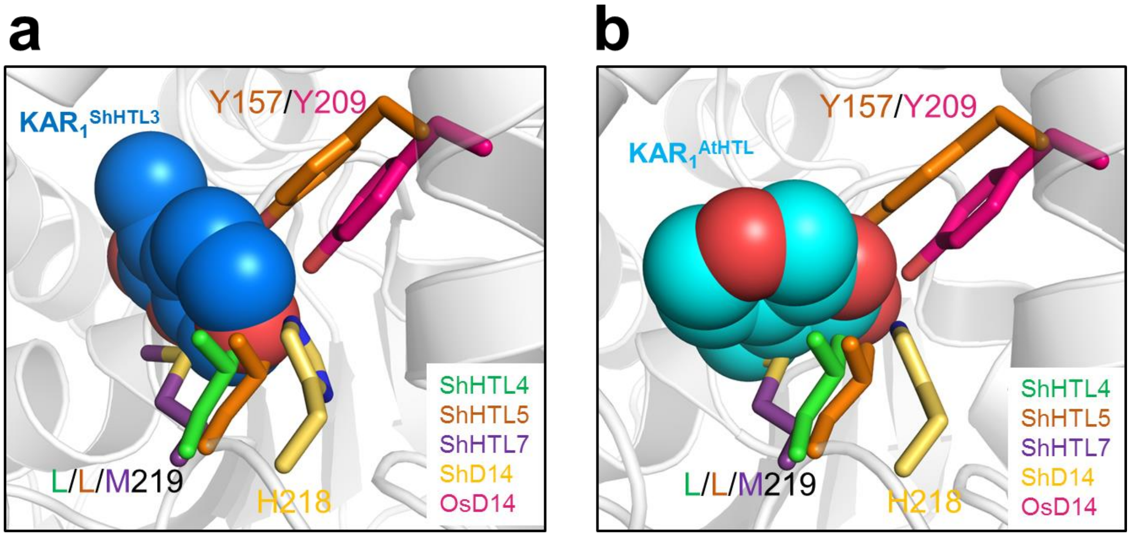

c

ShHTL7	T157	I218	M219
ShHTL5	Y157	K218	L219
ShHTL4	F157	M218	L219
ShHTL3	F157	M218	A219
ShHTL1	F157	L218	A219
AtHTL	F157	L218	A219
OsD14	Y209	V269	S270
ShD14	F158	H218	S219

Supplementary Figure 12. Alignments of KAR_1 in the pockets of ShHTLs and D14. (**a**–**b**) KAR_1 molecules from the ShHTL3-KAR_1 (**a**, PDB code: 5DNU; blue) and AtHTL-KAR_1 (**b**, PDB code: 4JYM; cyan) complex structures were modeled in the structures of ShHTL4 (green), ShHTL5 (PDB code: 5CBK, orange), ShHTL7 (purple), ShD14 (yellow) and OsD14 (magenta). (**c**) Residues involved in the KAR_1 binding of ShHTLs and D14. Residues causing steric hindrance are shaded green.

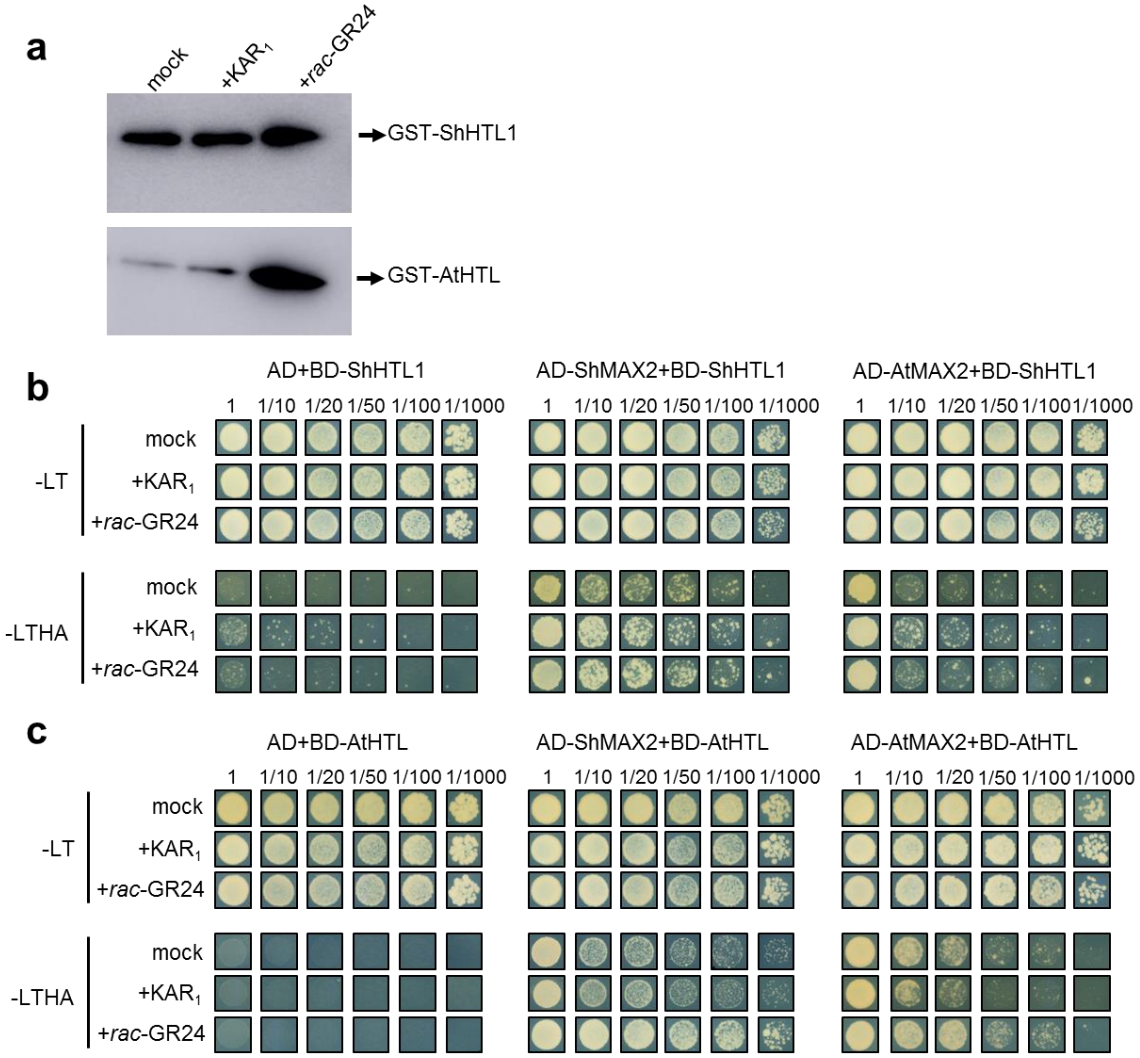

Supplementary Figure 13. Interactions between HTL and MAX2. (**a**) Interactions of ShHTL1-ShMAX2 and AtHTL-AtMAX2 were detected by western blotting. GST-fused ShHTL1 and AtHTL were detected using an anti-GST antibody, as indicated by the black arrows. (**b**) Interactions of ShHTL1 with ShMAX2 or AtMAX2 were detected by Y2H. A dilution series (1, 1/10, 1/20, 1/50, 1/100, 1/1000) of transformed yeast cells was plated on control medium (SD/-Leu/-Trp; -LT) and selective medium (SD/-Leu/-Trp/-His/-Ade; -LTHA) in the absence or presence of 5 μM KAR_1 or 5 μM *rac*-GR24. (**c**) Interactions of AtHTL with ShMAX2 or AtMAX2 were detected by Y2H.

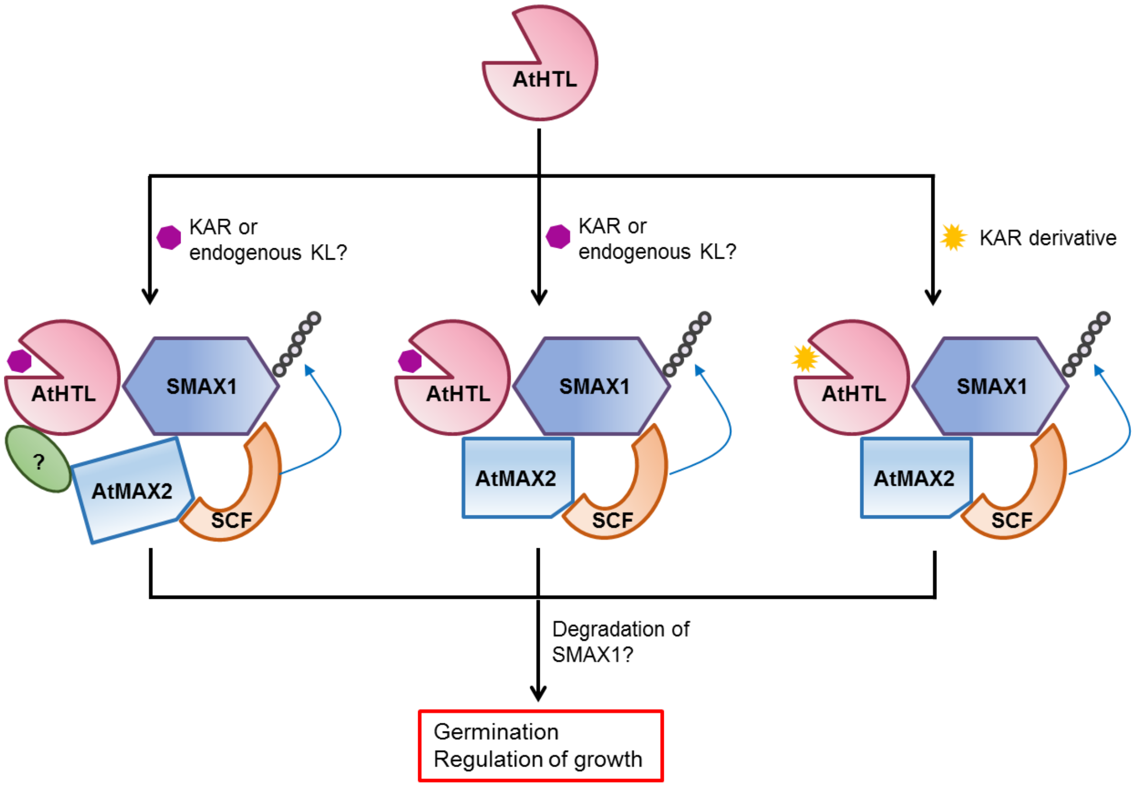

Supplementary Figure 14. Proposed AtHTL- and AtMAX2-mediated signaling pathways. This model is proposed based on the results of the structural analysis and biochemical assays in the present study. It is possible that AtHTL interacts with AtMAX2 via other unknown components (left) or that repressive SMAX1 (middle) is necessary to strengthen the AtHTL-ShMAX2 interaction. It is also possible that exogenous KAR is converted to an active form (KAR derivative) in the plant and induces the AtHTL-AtMAX2 interaction (right). Finally, similar to the SL signaling pathway, ubiquitin-mediated degradation of SMAX1 is induced to transduce KAR or KL signals.

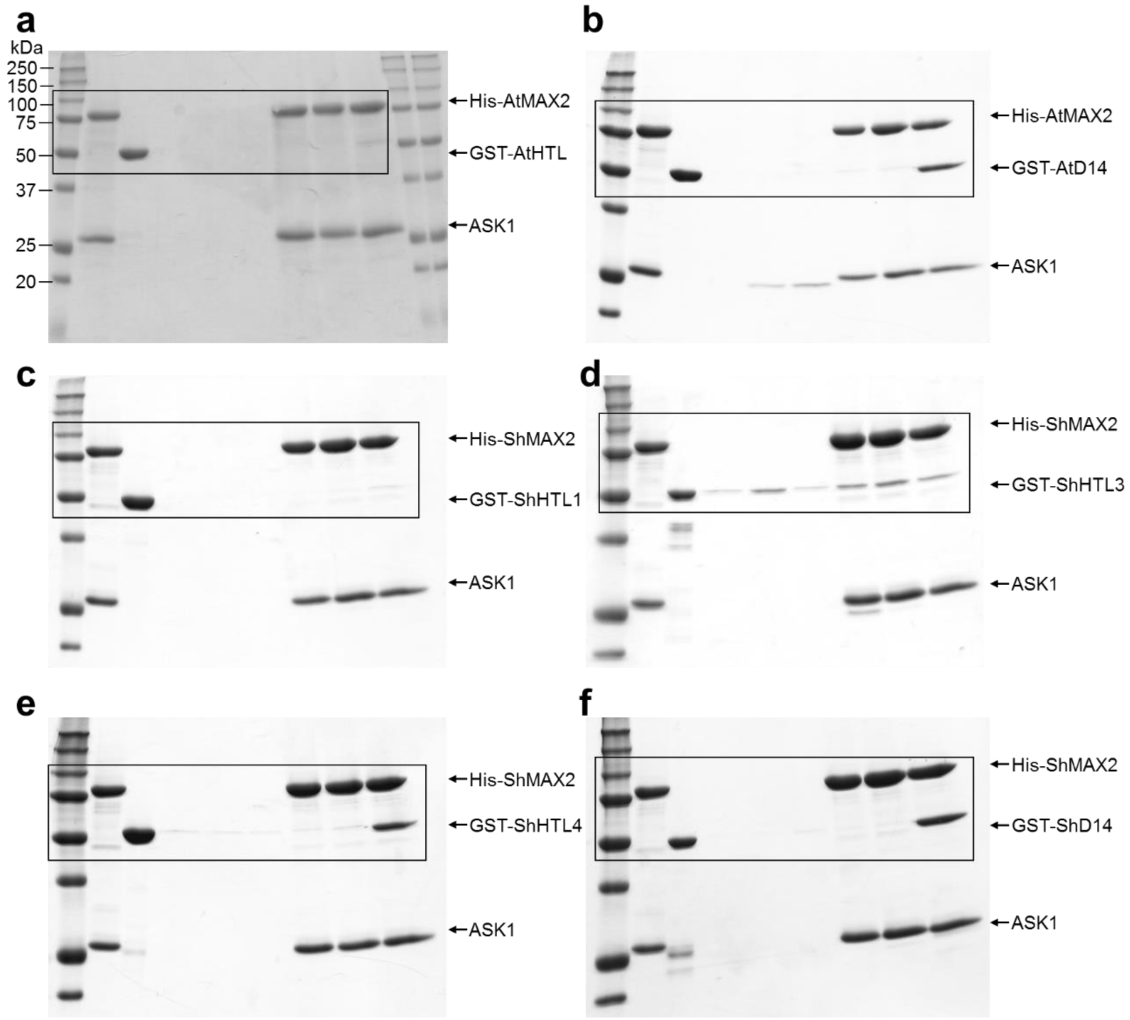

Supplementary Figure 15. Full scanned gels for pulldown assays in Fig. 5. (**a-b**) The full gels of the results of pulldown assays for interactions between AtMAX2 and AtHTL (**a**) or AtD14 (**b**) in Fig. 5b. (**c-f**) The full gels of the results of pulldown assays for interactions between ShMAX2 and ShHTL1 (**c**), ShHTL3 (**d**), ShHTL4 (**e**) or ShD14 (**f**) in Fig. 5c.

Supplementary References

1. Tamura, K., Stecher, G., Peterson, D., Filipski, A. & Kumar, S. MEGA6: Molecular Evolutionary Genetics Analysis version 6.0. *Mol. Biol. Evol.* **30**, 2725–2729 (2013).
2. Toh, S. *et al*. Structure-function analysis identifies highly sensitive strigolactone receptors in *Striga*. *Science* **350**, 203–207 (2015).
3. Conn, C.E. *et al*. Convergent evolution of strigolactone perception enabled host detection in parasitic plants. *Science* **349**, 540–543 (2015).
4. Thompson, J.D., Higgins, D.G. & Gibson, T.J. CLUSTAL W: improving the sensitivity of progressive multiple sequence alignment through sequence weighting, position specific gap penalties and weight matrix choice. *Nucl. Acids Res.* **22**, 4673–4680 (1994).
5. Robert, X. & Gouet, P. Deciphering key features in protein structures with the new ENDscript server. *Nucl. Acids Res.* **42**, 320–324 (2014).
6. Tsuchiya, Y. *et al*. Probing strigolactone receptors in *Striga hermonthica* with fluorescence. *Science* **349**, 864–868 (2015).
7. Cheng, Y.C. & Prusoff, W.H. Relationship between the inhibition constant (K_I) and the concentration of inhibitor which causes 50 per cent inhibition (I_{50}) of an enzymatic reaction. *Biochem. Pharmacol.* **22**, 3099–3108 (1973).
